# From I to we in small-scale fisheries communities

**DOI:** 10.1007/s40152-020-00204-z

**Published:** 2020-11-09

**Authors:** Svein Jentoft

**Affiliations:** Norwegian College of Fishery Science, Faculty of Bioscience, Fisheries and Economics, UiT-The Arctic University of Norway, 9037 Tromsø, Norway

I am immensely grateful to have had five prominent scholars share their insights regarding my MARE 2019 keynote address (slightly revised for publication), and to MAST for soliciting those responses. This is academia, as I have learned to know it, at its best. I find their remarks very illuminating. They are more supplements than negations. This must mean that we agree on why communities are important to the work and the well-being of people who draw livelihoods from “life below water.” I am not able, in a short reply like this, to comment on every point made by the commentators. They stand well on their own feet. I will therefore be selective and consider my comments as a continuation of a good conversation.

I concur with Lisa Campbell who says that we need to talk more rather than less about the role of communities in small-scale fisheries. This is not only because communities have functions that are essential for the sustainability of small-scale fisheries but also because they have values in themselves. They matter on their own terms. Fish is an important part of our food intake. If we are hungry, that is all we think of. But there is more to life than filling our bellies. People do not live on bread or fish alone. Unlike other primates, we search for meaning, but we do so in association with others.

Aristotle (in Politics) said that we are “by nature social animals,” dependent on each other to be well. For that, we need lasting social relationships, something that communities provide. Communities have to be there for us. Society is something that precedes the individual, he said. This is also how the sociologist Emile Durkheim later came to see the relation between the individual and society; individuals are a social construct. However, influenced by Berger and Luckmann (1967), I tried in my keynote to illustrate how the causal arrows between the individual fisher and the small-scale fishing community go in both directions; they construct each other mutually and continually.

Small-scale fisheries are typically community-based social systems, and can hardly exist otherwise. The solitary “bowler,” as Robert Putnam (2000) described him, should not be the role model on which to build fisheries policies and management systems. Neither should policy-makers see small-scale fisheries “like a state” (Scott 1998), i.e., imposing grand-scheme policies from the top down and afar as if small-scale fisheries are a unified sector, and just one particular type of fishing among others. Rather, they should see small-scale fisheries “like a community” (Dinya Karnard) with the contextual specificities, complexities, diversities, and dynamics of social life. This is why fisheries management must include a *phronetic* knowledge, as Aristotle called it, and not just science (Jentoft 2006).

## Finding communities

For communities to be sustainable, they need policy mechanisms that nurture the “We.” In his comment, David Symes mentions ITQs (Individual Transferable Quotas), which I also touched on in the talk. I have argued elsewhere that from a community perspective, it is neither the T nor the Q but the “I” in the ITQ system that is the most problematic. A community that is only an assembly of “I”s will have an atomistic fabric; people will be there for themselves and not for each other. Small-scale fisheries need social infrastructure. There is now plenty of evidence of ITQs hurting this quality of communities.

Behind the focus on the “I” rather than “We” is what social scientists recognize as methodological individualism. People are perceived as self-centered profit maximizers—of wealth, power, or prestige. The alternative view, which social scientists support, that communities are about “We” is rejected as “romanticism,” as Campbell points out. How idealizing the market is less romantic is not clear. There is nothing natural about markets or communities; they are social constructions. Communities obviously come with shortcomings, such as the tendency to limit individual freedoms, as discussed in my keynote. But so do markets, especially for those who find themselves on the losing side.

As both Lisa Campbell and Divya Karnad mention, small-scale fisheries have no clear definition. We obviously need a definition for counting and comparing. However, the enormous diversity and complexity of small-scale fisheries make it difficult to agree on a definition, which works everywhere. This is why it is lacking in the SSF Guidelines, and why the FAO has a very crude estimation of the scale of the sector globally. For policy and governance, definitions must be appropriate to context because local particulars and knowledge matters. Small-scale fisheries governance must follow the “dexterity principle” (Jentoft and Eide 2011).

I believe that the insistence on definitions runs the risk of derailing the understanding of small-scale fisheries. Bertrand Russel noted that we always need other words to define something, words which in themselves could need a definition. What are fishers and what is small anyway? We must agree on which approximations suffice in particular situations. We know small-scale fisheries when we see them. The missing definition of small-scale fisheries in the SSF Guidelines is no hindrance for starting to implement the Guidelines. Fisheries authorities know what SSF are and where to find them.

## The ambience of community

In my keynote, I discussed the moral aspects of small-scale fisheries communities. Communities typically come with a set of behavioral prescriptions, like the *Ubuntu* that Philile Mbatha refers to, which largely shapes how people interact with others and nature. But as I meant to illustrate with the Faroese chain dance, the experience of small-scale fisheries communities goes beyond that. We also have a feeling *of* community, but our sociological language is not rich enough to describe it. Try to define “love” and decide if the word captures what you felt when you had it, or have it. The words of social science do not capture the feeling. Shakespeare and Tolstoy do a better job than we social scientists can ever do.

I believe the same is true for communities. Sociological concepts fall short of fully expressing what communities mean to those who share them, and what they feel to have a community. We have tools to depict the structural and interactive attributes of communities, but our concept of community is not sufficient to capture its emotional qualities. To get the ambience of a small-scale fisheries community, watch Gershwin’s Porgy and Bess. Catfish Row, which is the imaginary scene for the opera, is a “complete” fishing community, working for its members on many levels. It is not just the music that is exhilarating but also the story about Catfish Row’s inhabitants’ life and love, their happiness and losses.

Therefore, we need a narrative that also includes the expressive sides of community. This, I submit, is what makes us convinced in our hearts that we are fighting the good cause when we try to convince policy-makers and managers about the need for small-scale fisheries communities to be part of the management equation. Divya Karnad observes that “policy makers around the world do not envision small-scale fisheries as a part of the future of any nation’s fisheries.” If that perception is widespread, social scientists have a job to do. We must then convey the message that community is not only about actions related to fishing and fisheries management, but to all other aspects of life.

This, I believe, is what Bonnie McCay is hinting at when she talks about the “comedy of the commons.” The same metaphor will also work for the community. The comedy comes alive in Catfish Row. In contrast to the misery that results from the tragedy of the commons, comedy embodies the prospect of happy outcomes. Fishers are not always in destructive competition but also in constructive cooperation, collective action, and solidarity. What nurtures one rather than the other is worth exploring. I submit that it is here that communities play a role. At their best, communities bring people together not just out of expedience because they have to cooperate, but also out of compassion for each other, because they care.

## Community as covenant

A person who listened to my presentation thought I was too brief about the role of religion, which I admit to. Social scientists have not overlooked religion. Indeed, Emile Durkheim argued that building community is religion’s most important function, that religion is less about believing than belonging. Communities are better off with a common morality—Durkheim used the term “moral community.” Loss of morality, religious or secular, may be what brings about community failure in sustaining the resource commons. Max Weber’s idea of “disenchantment of the world,” the deprivation of a genuine sense of community, erodes the spiritual “We,” and hence the community’s ability to create meaning, cohesion, stability, and social order. This is a different causality of the tragedy than that of Hardin. Without fishers’ sense of belonging to a moral “We,” fisheries become unmanageable, even with the presence of an external force.

I quoted Amitai Etzioni, who phrased sociology’s most fundamental insight as “*I* need a *We* to be *Me*,” a perspective he developed in his book “The moral dimension; towards a new economics.” (Etzioni 1988). Community is one of the most important “We” in our lives, which we need to be well. Jonathan Sacks argues that, as in marriage, the “We” works less as a contract than as a covenant. The former is about interest, whereas the latter is about identity. Thus, a covenant involves a deeper commitment than a contract does. “This is why contracts *benefit*, but covenants *transform*… Covenants heal what markets and states sometimes harm” (Sacks 2019: 327). A contract lasts as long as it is of mutual advantage to the parties, whereas covenant persists even with the sacrifice of freedoms. This, I hold, is how we should think of a well-functioning, small-scale fisheries community. It thrives when members feel morally rather than opportunistically committed, when guided by a sense of duty (deontology) instead of egoistic, utilitarian motives.

## Whose shoulders?

Philile Mbatha raises the important issue of western bias in our reading and referencing. We should obviously do more to become familiar with the broadest possible scholarship. There are, however, language problems, which a person with another mother tongue than English knows well. My own work would have been so much easier if everyone else were reading and writing in Norwegian, and I could do the same. Still, I believe that we should strive to know those whose shoulders we stand on as academics, regardless of who they are and where they come from, and even if they are long gone. We may think differently than they did, but our disciplines are stronger because of them.

A present bias brings the risk that we, without being aware of it, will reinvent the wheel. Scholars before us often explored issues that are still with us, such as what it means to be human, how we should live together, and what it means to be moral. We must never forget to ask these questions because they never go away. There are always new lessons to learn and new horizons to explore. Learning from others, whether from those of different language backgrounds, different cultural origins, or writers from the past, cannot be other than rewarding. Thus, as Philile Mbatha rightly points out, there is every reason to worry about the fact that we are missing important insights from non-White, non-Western, and female voices. We must make our disciplines and discourses more inclusive while resisting the tendency to impose Western perspectives on others. Learning must be interactive and a collective effort. The MARE (“People and the Sea”) conferences are a celebration of an epistemic community that involves people from all over the world. This is why I keep coming to them.

Nonetheless, the old thinkers I mentioned in my talk continue to be relevant. Like Bonnie McCay, I believe that classical social scientists and philosophers (some of whom are still with us) are worth consulting regardless of their cultural background, gender, race, political orientation, etc. We cannot fully get rid of the cultural baggage that we carry, and neither do I think that we should, as it would imply that we all think the same. We are also part of the world’s cultural richness, and part of global conversations.

## The romance of the community

Elinor Ostrom warned against the dangers of implementing policy fixes based on metaphors. They are too simplistic for a complex domain. They easily become self-fulfilling prophecies, as Robert Merton talked about, because what we believe becomes real when we act on it. This is the famous Thomas theorem.^1^ Small-scale fishing people are not the “rational egoists” that Hardin assumed, although if we lose community they may become like that.

There is also sufficient empirical evidence in support of David Symes when he makes modern quota fisheries management tools among the causes responsible for the demise of small-scale fisheries. Such management measures do not just have an impact on small-scale fishers but also transform them. Philile Mbatha lists a range of other sectoral activities threatening small-scale fisheries such as conservation measures, tourism, coastal or offshore mining, industrial fisheries, shipping, and aquaculture. She argues that in relation to these stakeholders, current policies alienate small-scale fishers from their rights. Small-scale fisheries then become a bitter endeavor—one of defeat.

If we are going to employ old Greek literary genres in our thinking about the commons and community, we should not only use tragedy, but also comedy, romance, and satire. These are different “modes of emplotment”, as Haydon White (2015) calls them.^2^ They are not only different ways of telling a story, but also of explaining a set of outcomes. Whereas comedy leads to reconciliation and tragedy to resignation, romance ends with a victory of the weaker party. For instance, in their struggle to sustain themselves or defend their beach, small-scale fisheries people are often up against actors more powerful than they are. For people in small-scale fisheries, life is like a classic romance, a struggle for justice. It contains the promise (but not a guarantee) of a positive outcome, as in Marx’s narrative where the proletariat will ultimately be victorious if they unite. This is also the optimistic narrative of the SSF Guidelines.

With Maarten Bavinck and Joeri Scholtens (2018), I have argued for a stronger research focus on fisheries as social struggle (2018), how small-scale fishers are involved in a fight for a better future, shaped in the emplotment of a romance. For sustaining small-scale fisheries, this is critical, as their interests are often in contradiction with those of other stakeholders. This is especially true now with the Blue Economy turning the coastal zone into a conflict zone. Social struggles are not just about scarce resources, as within Hardin’s narrative, but also about social justice, conflicts of interest, and a way of life.

## Re-embedding communities

As Divya Karnad points out, the COVID-19 crisis is taking its toll on small-scale fisheries communities. What makes local fisheries communities resilient and robust in a moral sense is also what makes people endure hardships, willing to sacrifice, and accept limits on their personal freedoms. In the current crisis, as she argues, these are essential albeit “invisible social capitals” that “can really make the difference between survival and death of fisheries.” No one saw the pandemic coming in June 2019 when I gave my keynote, but now it has changed how we think about the world and ourselves. Vaccines may help, but will not solve the problem if the moral fabric of the community dissolves. Well-integrated small-scale fisheries communities may cope better than fragmented communities. The current crisis provides an opportunity to give this thesis a test. Pandemics, as health crises, are essentially “wicked problems” (Rittel and Webber 1973; Jentoft and Chuenpagdee 2009) whose solution requires a social process and a rearrangement of social relations. How to combine compassion with the need for social distancing is a major challenge.

It is also from this perspective that we may see the FLAGs of the European Union that David Symes mentions. They are a test case of whether it is possible to re-embed a local, small-scale fishery that the neoliberal fisheries management system has dis-embedded. Will the FLAGs help communities move from “I” to “We,” with which they must to handle the COVID-19 crisis? How well they succeed is a matter for research. It will also provide answers to Lisa Campbell’s question on how we study social relations and interactions such that the results are relevant beyond the specific case. Divya Karnad posits that our research should follow a “bottom-up” approach. This is fine as long as we remember that case studies are not just about the case itself but what they are a case *of*.^3^

## The satire of the community

We need a critical and nuanced perspective on communities, including on their failures. It is then useful to employ the fourth of the classical literary genres, the “satire.” We do not have to like what we see, nor must we be quiet about what we do not like. We also should be self-critical about how we argue about things. We must be wary about what we say and how we say it, and remember that words may do more than describe; they can also be actions, as J.L Austin noted.

We must also explore the multiple discourses regarding the commons and the community. It can still be useful to play the Devil’s advocate, as the satirist would do. This was always my own approach to co-management. I am supportive of basic principles but choose to be critical of particular solutions. With co-management, things that can go wrong without thorough planning. The Devil is in the detailed designs and practices, not in the principles where God resides.

There are many ways of looking at fisheries communities and their commons. The doom and gloom of the tragedy is not their only prospect, as the different literary genres suggest. Criticism may leave a question of “so what?” We should not expect that other people have the answer to the questions we raise. Criticism is constructive if it points towards solutions, as when we shift metaphor from the tragedy to the comedy or the romance of the commons. This would allow us to see the potential of community and collective action, which are absent in Hardin’s narrative.

Notably, communities are rarely idyllic and harmonious, but have inbuilt contradictions, inequities, and “idiocies” (of rural life), as Marx called it in the Communist Manifesto. If we forget to be critical, including of our own perspectives and solutions, we do not do our job, and may be rightfully labelled naïve romantics. Metaphors and literary genres are just ways of looking at, thinking and talking about reality, not reality itself. Our work should be of help to those who struggle for justice. Foucault (2003) argued that our work “should be an instrument for those who fight, who resist and refuse… It doesn’t have to lay down the law for the law… It is a challenge to what is.”^4^

Still, I believe we are more effective if our criticism provides ideas about how things can be different, when we show that there are alternatives to the current order. We are not in the business of advocating panaceas, but we still have obligations to those who have legitimate expectations with regard to our contribution, like small-scale fishing people, showing them that things do not have to be the way they are—that there is nothing natural about the social systems that hold them down. Shifting metaphors can then be useful as a start.

## Footnotes

1 (https://www.oxfordreference.com/view/10.1093/oi/authority.20110803104247382).

2 White’s four literary genres inspired my book the Comedy of the Commons (in Norwegian (Jentoft 1998). The distinction between the genres can also be found in Frye (1990).

3 I owe this argument to Bent Flyvbjerg (2001).

4 https://www.goodreads.com/quotes/214758-the-necessity-of-reform-mustn-t-be-allowed-to-become-a
